# Chest Tubes and Pleural Drainage: History and Current Status in Pleural Disease Management

**DOI:** 10.3390/jcm13216331

**Published:** 2024-10-23

**Authors:** Claudio Sorino, David Feller-Kopman, Federico Mei, Michele Mondoni, Sergio Agati, Giampietro Marchetti, Najib M. Rahman

**Affiliations:** 1 Division of Pulmonology, Sant’Anna Hospital of Como, University of Insubria, 21100 Varese, Italy; sergio.agati@asst-lariana.it; 2 Section of Pulmonary and Critical Care Medicine, Dartmouth-Hitchcock Medical Center, Lebanon, NH 03766, USA; david.j.feller-kopman@hitchcock.org; 3 Respiratory Diseases Unit, Department of Internal Medicine, Azienda Ospedaliero Universitaria delle Marche, 60126 Ancona, Italy; f.mei@staff.univpm.it; 4 Department of Biomedical Sciences and Public Health, Polytechnic University of Marche, 60126 Ancona, Italy; 5 Respiratory Unit, ASST Santi Paolo e Carlo, Department of Health Sciences, Università degli Studi di Milano, 20122 Milan, Italy; michele.mondoni@unimi.it; 6 Pulmonology Unit, ASST Spedali Civili, 25123 Brescia, Italy; marchetti.giampietro@libero.it; 7 Oxford Centre for Respiratory Medicine, Oxford University Hospitals NHS Foundation Trust, Oxford OX3 9DU, UK; najib.rahman@ndm.ox.ac.uk; 8 Oxford Respiratory Trials Unit, University of Oxford, Oxford OX3 7LE, UK

**Keywords:** chest tube, pleural effusion, empyema, pneumothorax, drainage

## Abstract

Thoracostomy and chest tube placement are key procedures in treating pleural diseases involving the accumulation of fluids (e.g., malignant effusions, serous fluid, pus, or blood) or air (pneumothorax) in the pleural cavity. Initially described by Hippocrates and refined through the centuries, chest drainage achieved a historical milestone in the 19th century with the creation of closed drainage systems to prevent the entry of air into the pleural space and reduce infection risk. The introduction of plastic materials and the Heimlich valve further revolutionized chest tube design and function. Technological advancements led to the availability of various chest tube designs (straight, angled, and pig-tail) and drainage systems, including PVC and silicone tubes with radiopaque stripes for better radiological visualization. Modern chest drainage units can incorporate smart digital systems that monitor and graphically report pleural pressure and evacuated fluid/air, improving patient outcomes. Suction application via wall systems or portable digital devices enhances drainage efficacy, although careful regulation is needed to avoid complications such as re-expansion pulmonary edema or prolonged air leak. To prevent recurrent effusion, particularly due to malignancy, pleurodesis agents can be applied through the chest tube. In cases of non-expandable lung, maintaining a long-term chest drain may be the most appropriate approach and procedures such as the placement of an indwelling pleural catheter can significantly improve quality of life. Continued innovations and rigorous training ensure that chest tube insertion remains a cornerstone of effective pleural disease management. This review provides a comprehensive overview of the historical evolution and modern advancements in pleural drainage. By addressing both current technologies and procedural outcomes, it serves as a valuable resource for healthcare professionals aiming to optimize pleural disease management and patient care.

## 1. Introduction

Pleural drainage consists of inserting a flexible tube, called a chest tube or thoracostomy tube, through the chest wall into the pleural space. It is an essential procedure in both the diagnostic and therapeutic management of pleural diseases including pleural effusion, empyema, hemothorax, and pneumothorax. Instruments and techniques for pleural drainage have evolved significantly over time, reflecting advances in medical technology and a deeper understanding of pleural pathophysiology [1].

## 2. Historical Background

The concept of pleural drainage dates back to ancient times. Hippocrates (460–370 BC) is often credited with describing the first form of pleural drainage using hollow reeds to drain empyemas [2]. However, it was only in the 19th century that chest tube thoracostomy as we recognize it today began to take its current form [3].

### 2.1. Nineteenth Century Developments

Before the development of antibiotics, closed-space infections were almost exclusively the concern of surgeons, who generally approached them with early, aggressive, and open drainage. Little was known about the pathophysiology of the pleural space and open pneumothorax was considered the inevitable consequence of surgical evacuation except for cases in which the empyema caused adhesions between the visceral and parietal pleura, thus preventing lung collapse.

In 1871, British physician William Smoult Playfair devised subaqueous drainage to fully drain thoracic empyemas in children while preventing air from entering the pleural cavity [4]. Similarly, in 1875, German internist Gotthard Bülau introduced the closed drainage system using a siphon principle, which significantly reduced the risk of infection compared to open drainage [5]. Although Bülau’s technique was published in 1891, it was rarely used for several years.

### 2.2. Twentieth Century Advancements

In 1917–1918, during World War I, the influenza pandemic led to many cases of subsequent group-A streptococcal pneumonia and hemorrhagic pleural effusions in military camps, with very high mortality rates despite the use of open drainage. It was during this time that Evarts Ambrose Graham, a captain in the Army Medical Corps, was appointed to the U.S. Army Empyema Commission and began treating empyema successfully with closed drainage systems [6].

The World War II period saw further advancements in the emergency use of chest tubes for pleural diseases in soldiers. Indeed, the need for effective management of traumatic hemothorax and pneumothorax spurred innovations in chest drainage systems.

The introduction of plastic materials in the mid-20th century revolutionized chest tube design, making them more flexible and less prone to kinking. Closed thoracostomy and underwater seal drainage became the standard of care for blunt thoracic trauma and treatment in the Vietnam War [7].

In 1968, Heimlich designed a unidirectional valve which, when connected to the drainage tube, ensured the drainage of gas or fluid from the pleural space without backflow [8]. This system was sterile and disposable and had the advantage of allowing patient ambulation compared to bulky underwater drainage bottles.

## 3. Modern Equipment

Modern chest tube thoracostomy involves several key components (a tube, a drainage system, and a suction system) and techniques designed to improve patient outcomes, reduce clogging, and minimize complications.

### 3.1. Chest Tubes

Chest tubes are typically made from polyvinyl chloride (PVC) or silicone, and vary in size and design to suit different clinical scenarios. With the advancement of technology, various types of chest tubes have been developed. They can be straight, angled, spiral, or coiled at the end (referred to as “pig-tail”). They can drain through a central channel, with distal fenestrations at the tip and sides, or have several channels (i.e., a Blake drain, Ethicon, USA) to facilitate pleural fluid drainage. A radiopaque stripe aids tube recognition in chest X-rays and there is typically a marker that is a set distance to the most proximal drain hole (sentinel hole). Some tubes can have a double lumen for aspiration or infusion simultaneously [9]. It should be noted that the shape of the tube and their ability to “lock” are completely separate—and indeed, locking chest tubes should be avoided in pleural drainage due to the risk of intercostal artery laceration on removal.

The size of a chest tube is typically measured according to the French system, where it is expressed in “Ch” (Charrière, from the name of the creator) or more simply in “Fr” (French, from the country where Charrière lived) [10]. The value of Ch or Fr corresponds to the external circumference of the catheter, so the diameter in millimeters can be approximately calculated by dividing the “Fr” by 3. For example, a 12 Fr/Ch tube has an external diameter of about 4 mm.

Commonly “small-bore” chest tubes (SBCTs) range from 8 to 14 Fr and their insertion is less invasive. They are the tubes most commonly used to drain air (pneumothorax) as well as all different types of pleural effusion (including empyema and hemothorax), due to their high maneuverability, limited complications, and better tolerability by patients in comparison with large-bore chest tubes (LBCTs) [11]. 

**Figure 1:**
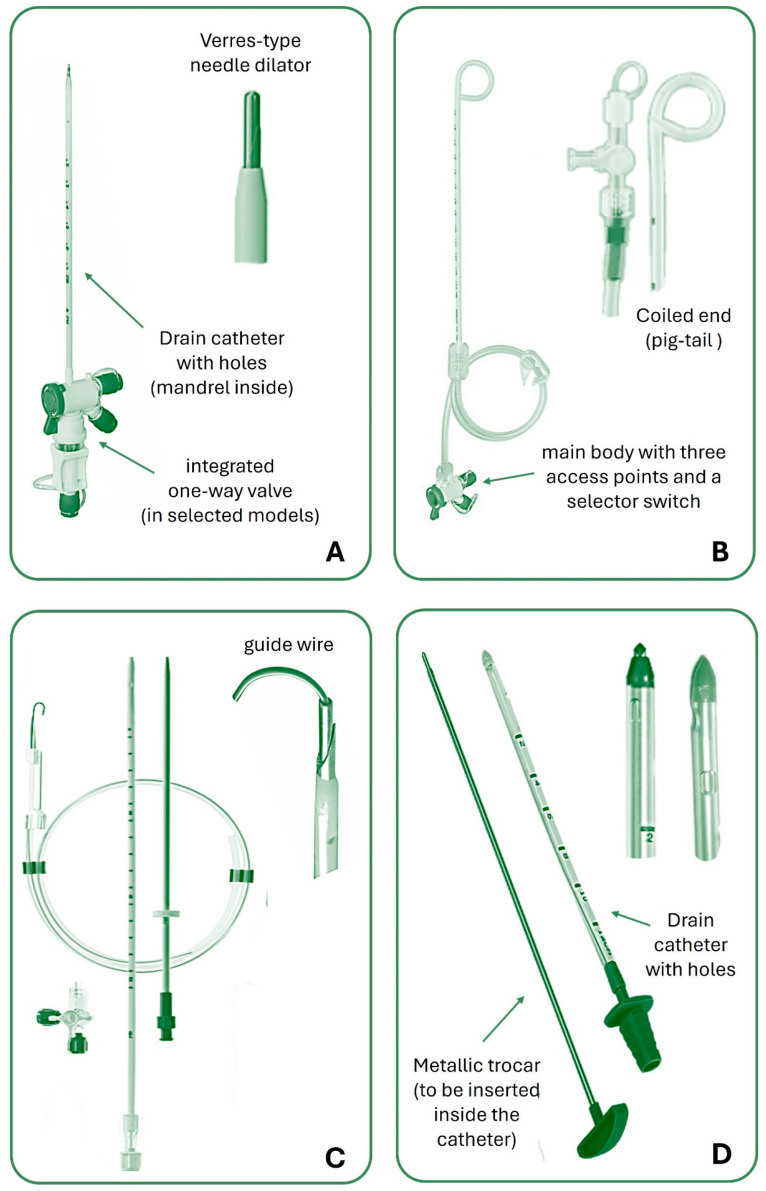
Main types of pleural drainage with details of the tips. (**A**): small-bore straight catheter with a Verres-type needle dilator; (**B**): small-bore pig-tail catheter; (**C**): small-bore straight catheter with guide wire for placement by means of the Seldinger technique; (**D**): large-bore catheter with trocar.

Among LBCTs (>14 Fr), those with a diameter between 16 and 24 Fr (sometimes referred to as medium-bore chest tubes) are often used for draining air or liquids including pus and blood, whereas 28 to 36 Fr tubes are usually reserved for draining thick fluids (hemothorax, empyema), especially in cases of severe trauma, need for rapid evacuation, or post-surgical drainage where there may be a large air leak. Larger tubes unavoidably lead to greater pain and complications. Figure 1 shows some commonly used types of chest tubes.

Note that large-bore tubes should not be inserted with a trocar due to the risk of tissue damage and complications. Blunt dissection is preferred, as it minimizes trauma and allows for safer placement compared to the guide wire technique, which is better suited to smaller catheters.

### 3.2. Chest Drainage Units

An adequate chest drainage system aims to remove pleural fluid and/or air, prevent their reflux into the pleural space, and restore negative pleural pressure (less than atmospheric pressure) to allow lung re-expansion [12]. 

Overall, a chest drainage unit (CDU) is a sterile, disposable device consisting of a flexible tube connected to one or more chambers that collect the fluid, to be positioned below the level of the chest tube insertion to allow the fluid to escape by means of gravity.

CDUs have evolved significantly since their introduction but essentially include one-way valves (Heimlich valve) or water-seal drainage systems to prevent the backflow of air or fluid into the pleural space.

**Figure 2:**
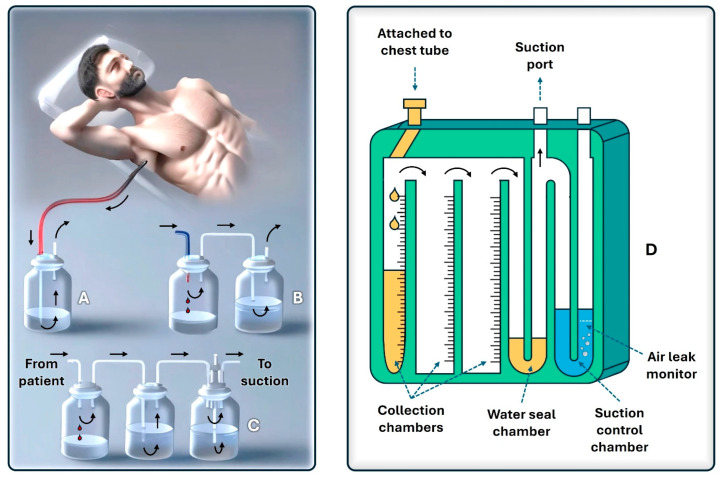
Exemplification of the classic underwater-seal chest drainage systems with one (**A**), two (**B**), and three (**C**) chambers, and a modern collection box (**D**).

An underwater-seal chest drainage system consists of a two- or three-chamber plastic unit with vertical columns displaying milliliter measurements (Figure 2). Their development stems from the original single-bottle system designed by Bülau, where a rigid straw, connected to the chest tube, entered the bottle and found itself with the tip immersed in saline solution. An opening with a one-way valve allowed air to escape and prevented pressure build-up in the system. However, the one-bottle system works well if only air exits the pleural cavity, whereas if a pleural effusion is drained, the fluid level in the bottle will increase and reduce the efficiency of removing additional air or fluid from the patient [12]. 

In two-bottle systems, the first bottle is responsible for collecting fluid, whereas the second bottle contains the water seal. They are preferred over the one-bottle system when large quantities of pleural liquid are drained, as fluid drainage does not affect the pressure gradient for further evacuation of fluid or air from the pleural space. Three-bottle systems have a third bottle or chamber, which is useful if suction is required.

All these chambers are currently integrated into modern, multifunctional, easy-to-manage boxes. Recently, smart digital drainage systems have been introduced, capable of recording the flows of evacuated air or liquid, monitoring the pleural pressure, and graphically reporting all the data [13,14]. 

### 3.3. Suction Systems for Pleural Drainage

The application of suction to pleural drainage systems can be useful in particular conditions not resolved by gravity drainage alone, to facilitate lung re-expansion and fluid or air removal. Data regarding the efficacy of suction following open or thoracoscopic lung surgery are controversial [15,16,17,18]. Similarly, data to support the hypothesis of a benefit in patients with pneumothorax are weak [19]. In theory, lung expansion obtained through external suction would allow the apposition of the visceral and parietal pleura to exert a compression effect on the area of a visceral pleural defect and consequently stop air leaks. However, excessive negative intrapleural pressure produced by suction may induce an increased airflow through the defect, especially in patients with non-expandable lung [20,21]. Traditional water-seal CDUs have been associated with a significantly shorter duration of postoperative air leak and chest drainage compared with continuous suction and digital drainages [22]. The application of suction should be avoided immediately after chest tube insertion as it may increase the risk of re-expansion pulmonary edema, particularly in young patients with complete pneumothorax or if the lung has been deflated for a prolonged time [23,24]. 

Water seals regulate the amount of suction through the height of a column of water in the suction control chamber. The suction control chamber is filled with water to the desired level. An external vacuum source generates negative pressure pulling air through the water column. The water column height resists this pull, thereby regulating the suction pressure to the set level. Wall suction provides consistent and adjustable suction pressure that is set by the depth of the column of liquid in the collection system and not by the suction read on the wall pressure gauge. With a 20 cmH_2_O water column in the suction control chamber, the maximum suction pressure exerted on the pleural space will be −20 cmH_2_O, regardless of the external vacuum source’s strength. This method ensures a consistent and precise level of suction. It requires regular checking and maintenance to ensure the water level is correct, as evaporation could alter the water level over time [25]. Newer systems use a ‘dry’ technique, where the amount of suction is applied by a setting on the drainage box. As with ‘wet’ systems, pressure to the patient can never be more negative than the pressure set on the chest drain.

Wall suction can be used in inpatients as it utilizes the hospital’s central vacuum system. The suction pressure is regulated through a control valve and applied to the pleural drainage system via tubing connected to the drainage chamber. In addition to being limited to hospital settings with central vacuum infrastructure, this system restricts patients’ mobility and involves a risk of applying excessive pressure if not properly regulated.

Portable suction devices can be used in both hospital and outpatient settings, particularly for ambulatory patients or those requiring home care. They use battery or electrical power to generate negative pressure, and are connected to the drainage system via tubing, providing adjustable suction settings. These devices enhance patient mobility and independence, although they can be less powerful than wall suction and require regular maintenance and battery charging.

Mechanical suction regulators are used in conjunction with water-seal or dry suction pleural drainage systems in hospitals to control the amount of negative pressure applied to the drainage system. They are connected between the wall suction source and the drainage system, ensuring that the pressure remains within a safe and therapeutic range, typically between −10 and −20 cmH_2_O. They require careful calibration and monitoring to ensure effective function.

## 4. Clinical Applications

Chest tubes are employed in various pleural diseases, each with specific indications and management protocols. Table 1 provides a summary of the indications for chest tube placement in both pleural effusion and pneumothorax, including relevant descriptions.

Table 2 summarizes the decision-making factors and properties of chest tube types, techniques of placement, and drainage systems, along with their advantages and drawbacks based on the patient’s clinical context. Each method and system present advantages and limitations, which should be weighed according to the patient’s condition, available resources, and operator expertise.

### 4.1. Pleural Effusion

Transudative effusions are typically managed medically, with chest tube drainage reserved for symptomatic relief or diagnostic purposes [26]. These effusions are usually the result of systemic conditions such as heart failure, liver cirrhosis, or nephrotic syndrome, where the underlying issue causes fluid to accumulate in the pleural space [27]. Treatment focuses on addressing the root cause, and in cases where significant symptoms, such as breathlessness, occur, a chest tube may be inserted to drain the fluid and provide relief [28]. Refractory symptomatic transudative pleural effusions despite maximal therapy constitute an indication for pleural drainage as an alternative to repeated thoracentesis [29]. 

Some observational evidence has supported the use of indwelling pleural catheters (IPCs) in such patients, whose main role lies in the symptomatic management of malignant pleural effusion. However, the data regarding transudates are not univocal and a recent randomized trial did not highlight a significant difference in breathlessness palliation over 12 weeks between IPC and standard care with therapeutic thoracentesis. Thoracentesis was associated with fewer complications, while IPCs reduced the number of invasive pleural procedures [30]. 

In patients with refractory hepatic hydrothorax waiting for liver transplantation or for whom it is contraindicated, transjugular intrahepatic portosystemic shunt (TIPS) placement represents the most useful treatment, although serial thoracenteses and insertion of an IPC represent possible second-line options [31,32]. 

Exudative effusions, on the other hand, are often associated with infections, malignancy, or inflammatory diseases, resulting from local factors affecting the pleura, such as increased capillary permeability, infection, or neoplastic pleura infiltration. Therapeutic drainage via chest tube is commonly required not only to relieve symptoms but also to obtain a sample for diagnostic analysis, which can guide further treatment [33]. 

In some instances, particularly when pleural effusion is recurrent, pleurodesis might be an option to reduce the risk of relapses [34]. Pleurodesis can be performed via the introduction of a sclerosing agent through the chest tube into the pleural space (“slurry technique”), causing adherences between the pleural layers, obliterating the space, and thus preventing the reaccumulation of fluid. This procedure is particularly beneficial in malignant pleural effusions or chronic conditions where repeated fluid buildup significantly impairs the patient’s quality of life. Pleurodesis can be achieved using various agents such as talc, autologous blood, tetracycline, doxycycline, or bleomycin, and can also be performed under direct visualization during medical thoracoscopy or video-assisted thoracic surgery (VATS) to ensure even distribution of the sclerosant and maximize efficacy (“poudrage technique”). Two large randomized trials have not shown a difference between slurry and poudrage [35,36]. 

The TIME1 randomized clinical trial demonstrated that larger chest tubes (i.e., 24F) are more efficient than smaller ones (12F) for inducing talc slurry pleurodesis in patients with malignant pleural effusion [37]. The authors would certainly recommend a tube size greater than 12F for pleurodesis attempts with talc due to issues with blockage in smaller tubes.

### 4.2. Complicated Parapneumonic Effusion or Empyema

A pleural effusion is defined as “complicated” when it becomes loculated, pH and glucose fall, and LDH increases. Pleural thickening and an effusion occupying more than half of the hemithorax may suggest a complicated parapneumonic effusion (CPE), though neither of these features is specific to CPE. In this stage, antibiotic therapy alone is generally not sufficient for healing. Empyema is a type of CPE characterized by the presence of frank pus in the pleural cavity or a positive Gram stain or culture. CPE and empyema require prompt drainage to increase the chances of resolving local infection, reduce the risk of further spread of microbes and sepsis, and prevent long-term sequelae such as fibrothorax [38]. The use of chest tubes in these settings is a well-established cornerstone of therapeutic intervention.

CPE can progress through three stages: exudative, fibrinopurulent, and organizing. In the early exudative phase, pleural fluid is free-flowing and can be easily drained by thoracentesis. As the condition advances to the fibrinopurulent stage, the fluid becomes more viscous due to fibrin deposition, often necessitating chest tube placement or adjunctive therapies such as tissue plasminogen activator (rTPA)/DNAse to facilitate drainage. In the organizing phase, where fibrous septations form, chest tube drainage alone may be insufficient, and additional interventions like medical thoracoscopy, VATS, or open decortication might be required [39,40]. 

The effectiveness of chest tube drainage is influenced by several factors, including the size and location of the effusion, the viscosity of the pleural fluid, and the presence of loculations. Consequently, careful patient selection and technique are paramount [41]. LBCTs of 20–28 French were generally preferred for their superior drainage capabilities in thick, purulent effusions. However, small-bore catheters (10–14 French) have gained popularity due to their less invasive nature and comparable efficacy in certain scenarios, particularly when combined with rTPA/DNAse therapy [42,43]. 

In addition to mechanical drainage, the role of intrapleural fibrinolytics and enzymatic debridement has been increasingly recognized, especially when simple drainage fails and the patient is not suitable for surgery. Agents such as rTPA combined with DNase can enhance drainage efficacy by breaking down fibrinous septations and reducing fluid viscosity, thereby improving outcomes in patients with loculated effusions [38]. 

### 4.3. Hemothorax

Pleural drainage, specifically the use of chest tubes, plays a critical role in the management of hemothorax, which is the accumulation of blood in the pleural cavity. The primary objectives of pleural drainage in hemothorax are to evacuate the blood, restore normal respiratory function, prevent clot formation, monitor for ongoing bleeding, and prevent long-term complications such as fibrothorax [44]. 

Hemothorax often results from traumatic injury, surgical complications, or spontaneous causes such as rupture of blood vessels in the pleura. Conservative treatment of occult hemothorax fails in over one patient out of five, and the presence of hemothorax greater than 300 mL and the need for mechanical ventilation predict the failure of conservative treatment and the need for a thoracostomy tube [45]. Immediate pleural drainage is essential to mitigate the risk of respiratory distress and to facilitate lung re-expansion. The placement of 28–32 French LBCTs has been historically recommended for initial management to ensure the effective evacuation of blood and clots. Recent evidence suggests that 14 Fr percutaneous pig-tail catheters can be equally as effective as 28–32 Fr tubes in patients with traumatic hemothorax or hemopneumothorax, resulting in reduced patient discomfort during and after insertion [46]. 

The initial volume of blood drained can provide crucial diagnostic information. Drainage of more than 1500 mL of blood upon chest tube insertion, or continued bleeding of more than 200 mL per hour over 2–4 h, often indicates the need for surgical intervention, such as thoracotomy, to control the source of bleeding. Moreover, in cases of retained hemothorax, where clotted blood remains in the pleural space despite initial drainage, early VATS has been shown to be effective. VATS allows for the direct visualization and removal of clots, reducing the risk of infection and fibrothorax [47].

The management of hemothorax with pleural drainage is associated with a significant reduction in morbidity and mortality when promptly and appropriately administered. Recent studies highlight the importance of early intervention and the use of adjunctive techniques such as VATS or thrombolytic therapy (tPA and DNase) in cases where conventional drainage fails to evacuate the hemothorax completely [48,49,50]. These advancements underline the evolving landscape of hemothorax management and the critical role of pleural drainage in improving patient outcomes.

### 4.4. Pneumothorax

Pneumothorax, characterized by the presence of air in the pleural cavity, can be classified as spontaneous or traumatic. Spontaneous pneumothorax can in turn be primary (occurring without any apparent underlying lung disease) or secondary (associated with pre-existing lung pathology). It has been suggested, though, that many patients with primary spontaneous pneumothorax actually have emphysema-like changes/pleural porosity that has not been identified by chest imaging, and the distinction between primary and secondary pneumothorax may not be as important [51]. 

Primary spontaneous pneumothorax (PSP) typically affects young, healthy individuals. It often results from the rupture of subpleural blebs or bullae, which are more common in tall, thin, young men. The most common causes of secondary spontaneous pneumothorax (SSP) are chronic obstructive pulmonary disease (COPD), cystic fibrosis, lung malignancy, or infections. These conditions compromise alveolar integrity, leading to air leakage into the pleural space. Traumatic pneumothorax results from blunt or penetrating chest injury (e.g., rib fractures, stab wounds). Iatrogenic pneumothorax is a subtype caused by medical procedures such as lung biopsies or central venous catheter placements.

Pneumothorax often requires chest tube placement for the evacuation of air and re-expansion of the lung (Table 1), while needle aspiration may be sufficient for small pneumothoraces. Chest tube insertion is highly effective in managing PSP. Success rates for lung re-expansion are high, typically around 80–90%. However, recurrence rates can be significant, with 23–50% of patients experiencing another episode.

In the last decade, the choice of whether to drain a PSP was mainly made based on the distance > 2 cm between the lung and the chest wall at the hilum (or 3 cm at the apices) on a posteroanterior chest X-ray [52]. However, many experts are adopting a more conservative approach in selected cases [24]. 

A recent randomized controlled study showed that 94% of patients with large but minimally symptomatic PSP treated conservatively achieved complete re-expansion within 8 weeks. The enrolled subjects had an SPS size ≥ 32%, corresponding to the sum of interpleural distances > 6 cm on an erect posteroanterior chest X-ray, according to the Collins method [53]. The success achieved with drainage was 98% but the difference was not statistically significant. Furthermore, patients treated conservatively experienced significantly lower rates of 12-month recurrence (8.8% versus 16.8% of patients undergoing thoracic drainage) [54]. 

Accordingly, the most recent British Thoracic Society (BTS) guideline for pleural disease emphasized that asymptomatic or minimally symptomatic patients with PSP pneumothorax may be managed conservatively without immediate invasive procedures [11,55]. 

In SSP, chest tube insertion is crucial due to potentially large and prolonged air leaks, and the increased risk of morbidity and mortality. Success rates are lower compared to PSP due to the underlying lung pathology. Persistent air leaks are more common, and additional interventions such as surgery or chemical pleurodesis may be required. In the presence of high surgical risk, maintaining the tube for a long time may be the only way to allow continuous evacuation of air from the pleural cavity and prevent tension pneumothorax [56]. 

Chest tube insertion is critical in managing traumatic pneumothorax, particularly when bilateral, when there is associated hemothorax or large air leaks. The management of pneumothorax has seen significant advances with the introduction of portable long-term air leak devices. These devices allow for safe and effective outpatient treatment, reducing hospital stays and healthcare costs while providing continuous monitoring of pleural air leaks. Moreover, they offer patients greater mobility and quality of life during the recovery process, making them an essential option in the management of both spontaneous and post-surgical pneumothorax [57]. 

Tension pneumothorax is an emergency condition where immediate chest tube insertion can be lifesaving by relieving pressure on the mediastinum and restoring cardiovascular stability.

## 5. Measures for Appropriate Chest Tube Placement

### 5.1. Insertion Site

Chest X-rays and computed tomography (CT) scans provide essential information for the diagnostic workup of pleural diseases that may require a chest drain. Thoracic ultrasound (TUS) has become the method of choice to define the indication for the procedure and choose the type of chest tube and the insertion site. Imaging examinations should always precede the placement of a chest tube unless the situation’s urgency and the setting do not allow it (for example, in the case of tension pneumothorax, especially in an out-of-hospital setting).

In adults, the fourth or fifth intercostal space (approximately at the level of the nipple in men) along the midaxillary line is commonly used as the chest tube insertion site to drain a pleural effusion. This corresponds to the “safe triangle” area, posterior to the pectoralis major muscle, and anterior to the latissimus dorsi muscle [58]. An incision 1 cm anterior to the midaxillary line appears to reduce the risk of damaging the peripheral nerves of the lateral thoracic wall [59]. Loculated pleural effusion may require different insertion positions, identified by ultrasound. Apical pneumothorax and tension pneumothorax are often drained through the second or third intercostal space at the midclavicular line. However, this site may be uncomfortable for the patient and leave an unsightly scar, so it should not be the first choice [60]. 

Occasionally two simultaneous or consecutive chest tubes may be necessary to effectively drain non-communicating infected fluid collections after attempted intrapleural fibrinolytics/DNAse. It is common practice to insert the chest tube using the so-called freehand technique, in which the doctor marks the entry point under ultrasound guidance and then performs the procedure immediately afterward without moving the patient.

### 5.2. Chest Tube Insertion Techniques

In most circumstances, nowadays chest drainage is performed at the patient’s bedside. Except for penetrating chest injuries, prophylactic administration of antibiotics ahead of chest tube placement is not required [61]. SBCTs can be inserted using the Seldinger technique, also known as the guidewire technique, or through an atraumatic stylet that introduces the drain in the pleural space without needing a guidewire. Modern kits for inserting SBCT have a Verres-type needle and an inner stylet with a dull tip to protect the lungs from injury. During insertion, the stylet’s blunt tip is pushed into the needle, exposing the cutting profile. After the needle reaches the pleural cavity, a spring pushes the atraumatic tip out to its previous position. These are the most widespread methods due to the ease of insertion and increased patient comfort [62]. 

The trocar technique consists of introducing the tube thoracostomy together with a trocar into the pleural space using strength. Its use is decreasing due to the greater risk of damaging the surrounding tissue, including blood vessels and lung parenchyma, leading to complications such as hemorrhage or lung injury. Thus, the authors would not recommend the use of the trocar. Blunt dissection, on the other hand, allows for a controlled and gradual separation of tissue layers, minimizing trauma. Unlike the guide wire technique, which is better suited for smaller bore catheters, blunt dissection ensures the safer placement of large-bore tubes in cases of significant pleural effusions or pneumothorax requiring rapid drainage [63]. 

To place a chest tube, the patient usually lies in the lateral or supine recumbent position. Once the intercostal space has been chosen, the skin must be disinfected and local anesthesia (usually Lidocaine) administered to the insertion site, up to the deeper tissues. Placement of the chest tube over an area of skin affected by infection or tumor infiltration should be avoided. The needle goes over the upper edge of the rib to reduce the risk of damage to the neurovascular bundle. Aspiration into the syrinx of air bubbles (in pneumothorax) or fluid (in pleural effusions) confirms that the needle has reached the pleural space. A small incision in the skin facilitates the introduction of the catheter [64]. 

To insert LBCTs, a blunt dissection is needed to reach the pleural space.

The tube should be directed posteriorly and downwards to drain a pleural effusion or toward the front and upward to remove air in pneumothorax. Once the tube is placed, it is sutured in place and connected to a drainage system with an underwater seal or suction.

### 5.3. Securing the Chest Tube

Anchoring a chest tube is essential to ensure its proper function and to prevent infections and dislodgement [65]. There is evidence that suturing chest tubes can lower the rates of their unintentional dislodgment outside the pleural cavity before a clinical decision to remove the drain (6.6% versus 14.8% of non-sutured drains) [66]. 

**Figure 3:**
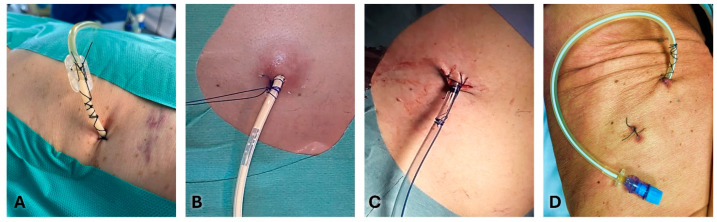
Different methods for anchoring a pleural drain. (**A**): Simple stitch and Roman sandal technique in a small-bore chest tube; (**B**): Simple stitch and tie of the drainage tube; (**C**): Purse-string sutures in a large-bore chest tube; and (**D**): Indwelling pleural catheter (IPC) secured by two simple stitches and a Roman sandal at the proximal end.

**Figure 4:**
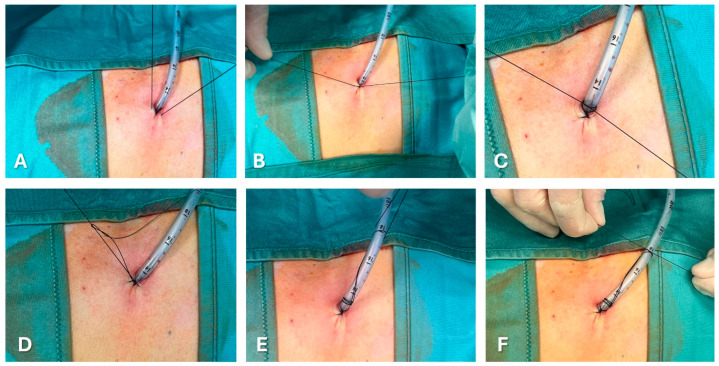
Progressive steps (from **A**–**F**) to secure a large-bore chest tube using the purse-string technique.

Figure 3 and Figure 4 show the most common methods for anchoring a pleural drain and the progressive steps to secure a large-bore chest tube using the purse-string technique. A recent multicenter trial compared a ballooned 12 Fr intercostal drain to a similarly sized tube secured with a single suture. The balloon integrated into the drain works like a bladder catheter which can be inflated with sterile water when it lies inside the pleural space to prevent it from slipping out [67]. The analyses of displacement rate showed a trend favorable to ballooned drain, although not statistically significant (3.9% vs. 10.1%). The control group’s displacement rate was less than expected in real-life practice, probably due partially to the high degree of utilization of ultrasound during the study.

The simple *Donati stitch* (horizontal mattress suture) uses a large-bore needle to place a suture through the skin, around the chest tube insertion site, and out through the skin again, forming a figure-of-eight or mattress suture.

In the *purse-string suture* technique, a suture is placed circularly around the chest tube insertion site. When tied, it cinches the tissue around the tube.

The *Roman sandal technique* is a common strategy to reduce dislodgement risk.

The suture thread is placed around the tube, crisscrossed, and tied in a manner resembling a Roman sandal’s lacing.

Tube securement devices, which adhere to the skin and grip the tube, providing an alternative to sutures, are commercially available. They offer a quick and often more comfortable way to secure the chest tube, with less risk of skin irritation and infection.

Finally, sterile adhesive dressings and tapes are used to anchor the tube to the chest wall. They reinforce the stability provided by sutures or securement devices, reducing movement and the risk of dislodgement. Usually, number 1 or 0 silk sutures are used for large bore tubes, and 00 for small bore tubes. When the incision has left space next to the drain, a second suture may be necessary to prevent the passage of liquid or air. The retaining stitches are commonly maintained 10–15 days after removing the chest tube.

## 6. Complications and Management

Despite advancements, chest tube placement is not without risks. Complications include tube malposition, infection, bleeding, organ injury, and re-expansion pulmonary edema. Preventative measures and prompt management of complications are critical. Table 3 shows the key complications that may arise during chest tube placement and provides succinct management strategies for each.

### 6.1. Preventative Measures

Rigorous adherence to sterile procedures minimizes the risk of infection, which is crucial given the direct access to the pleural space and the potential introduction of pathogens. This includes the use of full barrier precautions, proper skin antisepsis, and the sterile handling of equipment throughout the procedure. Ensuring that clinicians are adequately trained in both the technical and anatomical aspects of chest tube placement significantly reduces the risk of complications. This training should encompass a thorough understanding of chest wall anatomy, appropriate site selection for tube insertion, and the proper technique for securing and maintaining the chest tube. Furthermore, using imaging guidance such as ultrasound during insertion can enhance accuracy and safety. Regular competency assessments and continuing education help maintain high standards of practice. Additionally, the use of protocols and checklists can standardize procedures and reduce the likelihood of errors, contributing to improved patient outcomes and reduced complication rates [68]. 

### 6.2. Management of Complications

Chest tube management also involves meticulous care to prevent complications such as tube dislodgment, infection, and re-expansion pulmonary edema. Awareness of anatomical landmarks and the use of imaging guidance can reduce the risk of injuring the lung, diaphragm, or abdominal organs. Misplaced tubes may require repositioning (i.e., partial withdrawal) or replacement, often guided by imaging techniques. Antibiotic prophylaxis may be warranted in certain high-risk scenarios, and any signs of infection should prompt immediate evaluation and treatment. Regular monitoring of output, fluid characteristics, and imaging studies are essential to guide ongoing management and to determine the appropriate timing for tube removal. Moreover, when chest tubes are used to drain pus and other infectious materials, the viscosity of the fluid and the potential presence of fibrinous materials can increase the risk of occlusion. To prevent the blockage of drainage, as well as to cleanse the pleural cavity, continuous or intermittent flushing is recommended [19]. 

### 6.3. Training, Learning, and Practicing Chest Tube Management

Acquiring the skills and undergoing training to place and manage a chest tube is a multifaceted process that combines theoretical knowledge, simulation-based practice, and clinical experience. Initially, trainees should understand the indications, contraindications, and anatomical considerations of chest tube insertion. Comprehensive knowledge of pleural anatomy and pathophysiology is essential, as it underpins the decision-making process and procedural steps involved in chest tube placement. Theoretical learning is often supported by detailed guidelines and instructional videos.

Simulation-based training plays a critical role in skill acquisition, providing a risk-free environment for trainees to practice the insertion technique [69]. High-fidelity dummies and virtual reality simulators allow for repeated practice of needle insertion, guidewire manipulation, and catheter placement, helping trainees develop muscle memory and procedural confidence. Simulation also includes the use of bedside ultrasound, which is crucial for guiding the procedure and reducing complications such as organ puncture.

Hands-on clinical training, supervised by experienced physicians, is essential for translating simulation skills into real-world competence. During clinical rotations, trainees perform chest tube insertions on patients under direct supervision, receiving immediate feedback and guidance. This practical experience is invaluable for learning to manage complications, make quick and accurate decisions, and ensure patient safety.

Ongoing assessment and continuous professional development are integral to maintaining proficiency in chest tube management. Regular workshops, peer discussions, and advanced training courses help clinicians stay updated with the latest techniques and best practices. By integrating comprehensive theoretical education, hands-on practice, and continuous learning, clinicians are equipped to perform chest tube insertions safely and effectively, thereby improving patient outcomes in the management of pleural diseases.

A recent study investigated the state of training and experience among UK medical higher specialty trainees (HSTs) in performing Seldinger chest tube insertions in acute care settings [70]. The authors found that non-respiratory trainees had fewer procedures, and lower confidence and knowledge, posing a training and service delivery challenge with significant patient safety implications. Addressing these gaps is crucial for improving outcomes in pleural disease management.

## 7. Conclusions

Chest tube thoracostomy and pleural drainage remain cornerstone interventions in the management of pleural effusion and pneumothorax. The evolution from ancient techniques to modern, sophisticated systems underscores the importance of continuous innovation and education in this field. Recent advances include the use of smaller bore catheters, which are less invasive and have shown similar efficacy to traditional chest tubes in select patients. Additionally, digital chest drainage systems offer real-time monitoring of intrapleural pressures and air leaks, enhancing clinical decision-making. Ongoing research and technological advancements hold the promise of further improving the efficacy and safety of these critical procedures, ultimately enhancing patient outcomes in pleural disease management.

## Figures and Tables

**Table 1:** Main indications for chest tube insertion.

Condition	Indication	Description
**Pleural** **Effusion**	Large Pleural Effusion	Significant accumulation of fluid causing respiratory distress or hypoxemia
	Complicated Parapneumonic Effusion/Empyema	Presence of infected fluid or pus in the pleural space (empyema) requiring drainage
	Malignant Pleural Effusion	Symptomatic effusion associated with malignancy, especially if recurrent. A chest tube may allow chemical pleurodesis if significant contact between the visceral and parietal pleura is achieved.
	Hemothorax	Accumulation of blood in the pleural space, often due to trauma or post-surgical complication
	Chylothorax	Accumulation of lymphatic fluid in the pleural space, often due to thoracic duct injury
	Pleural Effusion with unclear etiology	Diagnostic purpose when the cause of effusion is unknown and requires analysis of pleural fluid
	Post-Surgical or Post-Procedure	Prevention or management of fluid accumulation after thoracic surgery or procedures
**Pneumothorax**	Large Pneumothorax	Significant accumulation of air causing respiratory distress or hypoxemia
	Symptomatic Pneumothorax	Presence of symptoms such as severe breathlessness, chest pain, or hypoxemia
	Tension Pneumothorax	Medical emergency with hemodynamic instability requiring immediate decompression (simple needle aspiration is usually the first procedure in life-threatening situations)
	Recurrent Pneumothorax	Repeated episodes of pneumothorax after initial conservative management (thoracoscopy is another option)
	Secondary Pneumothorax	Pneumothorax in the presence of underlying lung disease with a higher risk of complications
	Traumatic Pneumothorax	Pneumothorax due to chest injury with significant air leaks or associated hemothorax

**Table 2:** Pros and cons of different types of chest tubes, insertion techniques, and chest drainage units (CDU) based on the patient’s clinical picture.

Category	Type	Pros	Cons	Best Suited For
**Chest Tubes**	Small Bore (≤14 Fr)	- Minimally invasive - Less painful - Lower infection risk	- Limited drainage capacity - Potential clogging	- Pneumothorax - Malignant effusions with low fluid volume
	Large Bore (>14 Fr)	- Effective for large volumes of fluid/air - Efficient drainage in hemothorax/empyema	- More painful - Higher risk of infection - Requires procedural expertise or surgeon	- Hemothorax - Empyema - Traumatic pneumothorax
	Pig-tail Catheter	- Minimally invasive - Less painful - Coiled design reduces tissue trauma - Good for long-term use	- Can kink or clog - Limited use in viscous fluids - Less effective in large pneumothoraces	- Malignant pleural effusions - Spontaneous pneumothorax
**Insertion** **Techniques**	Verres-type Needle	- Rapid deployment - Useful in emergencies - Minimally invasive	- Limited control over placement - Higher risk of misplacement or injury to lung parenchyma	- Tension pneumothorax (emergency)
	Seldinger Technique	- Greater precision with wire guidance - Lower complication rates - Can be used for small bore tubes	- More time-consuming - Requires skilled operator - More equipment needed	- Pneumothorax - Non-emergency situations
**Chest** **Drainage** **Unit** **(CDU)**	Bag	- Portable - Simple and cost-effective	- Limited suction control - Inadequate for continuous monitoring	- Short-term use - Transport of stable patients
	One-Chamber	- Simple design - Low-cost	- Limited monitoring capability - No automatic fluid separation	- Small pneumothorax - Minor effusions
	Two-Chamber	- Allows air-fluid separation - Moderate monitoring capabilities	- Limited to specific clinical conditions - Intermediate suction control	- Small to moderate effusions
	Three-Chamber	- Provides air-fluid separation - Allows continuous suction with improved monitoring	- Bulky - Less portable - Requires proper setup and training for use	- Large effusions - Continuous drainage (e.g., hemothorax, empyema)
	Digital	- Real-time monitoring - Automated pressure regulation - Improved patient outcomes	- Expensive - Requires electricity - Not available in all settings	- Post-operative care - Complex pleural effusions or air leaks

**Table 3:** Potential complications during chest tube insertion and their management.

Complication	Description	Management
**Malposition**	Tube placed outside the pleural space or in an incorrect position	Immediate imaging (US/X-ray/CT) Repositioning or replacement
**Infection**	Pleural cavity or insertion site infection. The risk is higher for long-term chest tubes.	Aseptic technique Antibiotics if signs of infection Check for the appearance of pus or laboratory signs of infection
**Bleeding**	Injury to intercostal vessels or lung parenchyma	Apply direct pressure Surgical consultation if severe
**Re-expansion** **Pulmonary Edema**	Rapid lung re-expansion after drainage of large effusions/pneumothorax	Limit drainage frequency Provide oxygen Diuretics if needed
**Persistent Air Leak**	Continuous air bubbling indicating air leak	Clamp test to locate leak Surgical consultation if persistent
**Subcutaneous Emphysema**	Air leakage into subcutaneous tissues	Ensure proper tube placement Adopt suction or repositioning tube
**Tube Occlusion**	Blockage of the chest tube (e.g., clots, fibrin, thick fluid)	Flush or replace tube Monitor output closely

## Data Availability

No new data were created or analyzed in this study. Data sharing is not applicable to this article.
